# Antarctic *Sphingomonas* sp. So64.6b showed evolutive divergence within its genus, including new biosynthetic gene clusters

**DOI:** 10.3389/fmicb.2022.1007225

**Published:** 2022-11-18

**Authors:** Kattia Núñez-Montero, Dorian Rojas-Villalta, Leticia Barrientos

**Affiliations:** ^1^Extreme Environments Biotechnology Lab, Center of Excellence in Translational Medicine, Universidad de La Frontera, Temuco, Chile; ^2^Biotechnology Research Center, Department of Biology, Instituto Tecnológico de Costa Rica, Cartago, Costa Rica; ^3^Scientific and Technological Bioresource Nucleus (BIOREN), Universidad de La Frontera, Temuco, Chile

**Keywords:** natural products, antibiotics discovery, extreme environments, Antarctic, genome mining

## Abstract

**Introduction:**

The antibiotic crisis is a major human health problem. Bioprospecting screenings suggest that proteobacteria and other extremophile microorganisms have biosynthetic potential for the production novel antimicrobial compounds. An Antarctic Sphingomonas strain (So64.6b) previously showed interesting antibiotic activity and elicitation response, then a relationship between environmental adaptations and its biosynthetic potential was hypothesized. We aimed to determine the genomic characteristics in So64.6b strain related to evolutive traits for the adaptation to the Antarctic environment that could lead to its diversity of potentially novel antibiotic metabolites.

**Methods:**

The complete genome sequence of the Antarctic strain was obtained and mined for Biosynthetic Gene Clusters (BGCs) and other unique genes related to adaptation to extreme environments. Comparative genome analysis based on multi-locus phylogenomics, BGC phylogeny, and pangenomics were conducted within the closest genus, aiming to determine the taxonomic affiliation and differential characteristics of the Antarctic strain.

**Results and discussion:**

The Antarctic strain So64.6b showed a closest identity with *Sphingomonas alpina*, however containing a significant genomic difference of ortholog cluster related to degradation multiple pollutants. Strain So64.6b had a total of six BGC, which were predicted with low to no similarity with other reported clusters; three were associated with potential novel antibiotic compounds using ARTS tool. Phylogenetic and synteny analysis of a common BGC showed great diversity between Sphingomonas genus but grouping in clades according to similar isolation environments, suggesting an evolution of BGCs that could be linked to the specific ecosystems. Comparative genomic analysis also showed that Sphingomonas species isolated from extreme environments had the greatest number of predicted BGCs and a higher percentage of genetic content devoted to BGCs than the isolates from mesophilic environments. In addition, some extreme-exclusive clusters were found related to oxidative and thermal stress adaptations, while pangenome analysis showed unique resistance genes on the Antarctic strain included in genetic islands. Altogether, our results showed the unique genetic content on Antarctic strain Sphingomonas sp. So64.6, −a probable new species of this genetically divergent genus–, which could have potentially novel antibiotic compounds acquired to cope with Antarctic poly-extreme conditions.

## Introduction

From 2000 to 2015, antibiotic consumption increased by 65% (Klein et al., 2018). In addition, the proportion of the non-prescription supply of antibiotics was 62% from 2000 to 2017 (Auta et al., 2019). The misuse and overuse of antibiotics increased the mortality of common infections due to resistant strains of microorganisms, resulting in 1.27 million deaths globally attributed to antibiotic resistance in 2019 (Murray et al., 2022). Hence, the antibiotic resistance crisis is a major human health problem and is growing every year.

In this sense, developing new compounds with antimicrobial activity and novel mechanism of action is a main concern in the scientific community. Many studies have focused on using metals nanoparticles (e.g., silver) or a combination of nanomaterials and antibiotics to enhance their antimicrobial activity (Liao et al., 2019; Hochvaldová et al., 2022). Another recent approach is the use of predatory bacteria to kill pathogen bacteria, a technique that demands further investigations (Atterbury and Tyson, 2021). However, exploring secondary metabolites from microbes with antibiotic activities remains the primary source of research (Newman and Cragg, 2016).

Actinobacteria have been extensively explored for antibiotic compounds, with great variety and biotechnological potential (Genilloud, 2017; Jagannathan et al., 2021). Regardless of the promising results of antimicrobial compounds, the chances of discovering new metabolites from actinobacteria are low, forcing to explore new sources for bioprospecting (Bredholt et al., 2008). Some studies have suggested that proteobacteria might be a better target than actinobacteria for screening antimicrobial compounds (Al-Amoudi et al., 2016).

Proteobacteria is a diverse phylum of Gram-negative bacteria. Many natural products with medical relevance are produced by proteobacterial strains, such as the previously reported didemnins and thalassospiramides, showing medical relevant activities (Timmermans et al., 2017). Also, species of that phylum have been highlighted for its biotechnological potential for bioremediation and pollutants degradation, including *Alcanivorax* and *Marinobacter* strains (Chernikova et al., 2020). Moreover, the proteobacterial genus, *Pseudoalteromonas,* can produce a bioactive compound called thiomarinols, which is used against the multidrug-resistant pathogen *Staphylococcus* (Fukuda et al., 2011).

Bacteria from extreme environments (extremophiles) have recently been recommended for its predicted untapped variety of antimicrobial compounds, representing another focus of research for new antibiotics (Danilovich et al., 2018; Babich et al., 2021). Accordingly, extremophile proteobacteria have also been described to produce a great variety of specialized metabolites (Puri, 2019). Antarctic proteobacteria have shown antimicrobial activity against *Candida albicans* and *S. aureus*, both pathogenic, including bacteria from the *Sphingomonas* genus (Lee et al., 2012). This genus is best known for its ability to degrade organic pollutants, such as pharmaceutical and industrial residues (Aulestia et al., 2021; Zhang et al., 2022; Zhou et al., 2022). Nonetheless, antimicrobial activity has also been reported from species of the *Sphingomonas* genus, such as *S. molluscorum* (Romanenko et al., 2007; Lee et al., 2012).

In previous work, we reported an Antarctic *Sphingomonas* strain So64.6b with antibiotic activity in response to elicitation treatments (Núñez-Montero et al., 2020). Moreover, during elicitation treatments, it exceeded the number of metabolites capable of producing under regular conditions (Núñez-Montero et al., 2020). It was hypothesized that adaptation to the Antarctic might drive an untapped diversity of specialized metabolites; nonetheless, there is no evidence of a correlation between the bioactivity or production of new compounds with the adaptation to the extreme environments. Therefore, in this work we aimed to determine the genetic evolutive traits of *Sphingomonas* sp. So64.6b that might be associated with adaptation to the Antarctic environment and that could lead to its diversity of antibiotic metabolites.

## Materials and methods

### Antarctic bacterial strain and culture

An Antarctic bacteria strain (ID So64.6b) previously isolated from soil on Agar M1 (peptone 2.0 g/l, yeast extract 4.0 g/L, starch 10.0 g/L and agar 18 g/L; Lamilla et al., 2017) was used in this study. For storage, the bacterium was cultured in 3 ml of International *Streptomyces* Project-2 medium (ISP-2; yeast extract 4.0 g/L, malt extract 10.0 g/L, glucose 4.0 g/L, and agar 18 g/L) and cryopreserved at -80°C with 20% glycerol. Culture for DNA extraction was obtained by inoculating the isolated bacterial strain on the ISP-2 agar at 15°C for 7 days.

### DNA extraction and genome sequencing

Genomic DNA extraction was performed using the UltraClean Microbial DNA Extraction Kit (MoBio Laboratories, Carlsbad, USA). DNA quality was assessed by fluorescence quantification using the QuantiFluor® ONE dsDNA System kit (Promega) on a Quantus device (Promega). Also, integrity was verified by 1% agarose gel and purity according to 260/280 and 260/230 absorption ratios. The sample was divided into two and sequencing using Illumina and Oxford Nanopore Technologies (ONT) technologies. The Illumina library was prepared with 2x150bp fragments using the Nextera XT DNA Sample Prep kit (Illumina, San Diego, CA) and sequenced on an Illumina MiSeq platform. The ONT library was prepared using the Rapid Sequencing kit SQK-RBK004 and sequenced on the MinION platform of the Extreme Environments Biotechnology Lab (Universidad de La Frontera, Chile), according to the manufacturer’s recommendations and using the MinKNOW software v.4.0.20. The basecalling of the ONT reads was performed with Guppy v3.1.5 software.

### Whole genome assembly

Assembly was performed using the two data libraries (i.e., Illumina and ONT). The quality of Illumina reads was verified by FastQC (Andrews, 2010), and fastp (Chen et al., 2018) was used to filter poor-quality reads using default parameters. The quality of ONT reads was verified with nanoPLOT (De Coster et al., 2018). Adapter and low quality reads (phred >10, size >5,000 bp) were removed with Porechop and NanoFilt (De Coster et al., 2018), respectively. *De novo* assembly was performed using the Unicycler software v.0.4.8 (Wick et al., 2017) with default parameters for hybrid assembly with short + long reads. The whole genome results of this study were deposited in the DDBJ/ENA/GenBank database under the accession CP048817.

### Genome-based taxonomic and phylogenetic analysis

Whole genome taxonomic identity was confirmed using Average Nucleotide Identity (ANI) values. The ANI value was calculated among the available *Sphingomonas* representative complete genomes on NCBI database (×49) using Pyani v0.2.10 software in ANIb mode (Pritchard et al., 2016). The reference genome with highest ANI value was used for DNA–DNA hybridization (DDH) estimation values, which was computed using the genome-to-genome distance calculator (GGDC v3; Meier-Kolthoff et al., 2022). In addition, phylogenetic distances between the closest taxonomic species were also determined by constructing the core proteome of the available representative complete genomes on the genus *Sphingomonas* (×49). First, core proteins were obtained with the web server M1CR0B1AL1Z3R^1^ (Avram et al., 2019) using an 80.0% cutoff value for the minimum identity of proteins found in all compared genomes. Next, an unrooted phylogenetic construction of the core proteome was performed with a maximum likelihood algorithm using RAxML (Stamatakis, 2014) based on the inferred proteome alignment. Phylogenetic tree visualization was performed with the iTOL v3 tool (Letunic and Bork, 2016).

Finally, for same-species strains, the single enriched functions for each strain were studied based on Gene Ontology using the OrthoVenn2 server with default parameters (accessed on 12/15/2020; Xu et al., 2019). Protein sequences obtained from Prokka annotation (Seemann, 2014) were used as input. Briefly, similar proteins are obtained by all-against-all alignment with DIAMOND v0.9.24 (Buchfink et al., 2014) with a threshold of e-value = 0.05. Then, clusters are generated with MCL (inflation value = 1.5) and gene ontology (GO), and enrichment is assigned by similarity with the UniProt protein database (Apweiler et al., 2004). GO enrichment analysis computed the *p*-values for GO terms in a clusters overlapping using a hypergeometric distribution with the variables *N* = total count of all GO terms, *M* = count of GO terms in the overlap, *n* = total count of a GO term and *x* = count of the GO term that overlap on the following formula:


$$P=\frac{\left(\frac{M}{x}\right)\left(\frac{N-M}{n-x}\right)}{N/n}$$

### Genome mining of biosynthetic gene clusters

Genetic elements associated with the production of secondary metabolites (BGCs) on the So64.6b genome were identified using the antiSMASH v.5.1.0 tool (Blin et al., 2019) with default parameters and selecting all prediction features. Additionally, structure prediction of BGCs was performed on PRISM 3 (Skinnider et al., 2017). The closest known BGCs were assigned when matches were found compared to the MiBiG Cluster Repository of Known Biosynthetic Gene Clusters.

Clusters associated with the production of potentially novel antibiotic secondary metabolites were predicted with ARTS v2.0 (Mungan et al., 2020) with default parameters. A search for known resistance elements is performed by comparing with the curated databases: ResFams, The Comprehensive Antibiotic Resistance Database (CARD), The LACtamase Engineering Database (LACED), and The Jacobi and Bush Collection. In addition, essential genes are identified with TIGRfam Equivologs as the core genes from a reference set of genomes and classified according to: (a) rare duplication (possible resistant target genes), defined as all essential genes that exceed the median and standard deviation thresholds of the sum of essential genes identified in the bacterial family; (b) acquisition by horizontal transfer when comparing the topology of core gene phylogeny against species tree; and (c) their proximity to the BGCs identified with ANTISMASH 3 software (Mungan et al., 2020). This allowed the prediction of the mode of action of encoded compounds of an uncharacterized BGC based on the identification of resistant target genes (Mungan et al., 2020), improving the selection of the strains with the highest potential for antibiotic metabolites discovery.

### Comparative genomic analysis

#### Selection of genomes data Set

The maximum number of representative complete genomes of the taxonomically closest species were obtained from the NCBI database (accessed December 20, 2021) and subsequently used for genomic and phylogenetic comparison of the selected strains. Only representative genomes from the closest species were included for pangenome comparative analysis (×49). The chosen reference genomes were simultaneously re-annotated using Prokka 1.12 (Seemann, 2014).

#### BGC evolutionary comparison across the closest taxon

To understand the evolutionary relationships between secondary metabolites related to the production of potentially novel antibiotic compounds, a phylogenetic analysis of the main synthases present in most of the genomes of the dataset was performed. To do this, the BGC of interest with an analogous antimicrobial production cluster (using a BGC from MiBIG as reference) was selected. The 10 clusters with the highest similarity belonging to taxonomically close species (same genus) were chosen for this evolutionary analysis. The BGCs were predicted from each genome using antiSMASH 5.0 as previously described. The output .gbk files were used to determine genomic variations in major biosynthetic enzyme contiguity with CORASON platform of the BiG-SCAPE/CORASON tool^2^. CORASON employs a phylogenomic approach to elucidate evolutionary relationships between gene clusters by calculating high-resolution multi-locus phylogenies of BGCs within and across gene cluster families (GCFs); moreover, it allows to comprehensively identify all genomic contexts in which gene clusters of interest (‘subclusters’ within larger BGCs) can be found (Navarro-Muñoz et al., 2020).

Briefly, a conserved core of the reference BGC (from MiBIG) within the genomic database is identified using an all-versus-all algorithm between each gene set to remove the paralogs and retain only the true orthologs. As a primary biosynthetic enzyme, a reference gene is selected to ensure that at least one element will be present in the conserved core and is also used to align the BGC variations in the graphical output visually. Core sequences are identified by the longest common subcluster between the PFAM of a pair of BGCs, in this case in comparison with the cluster from the Antarctic *Sphingomonas*. For each BGC, the core sequences are concatenated and then aligned using MUSCLE v.3.8.31. The alignments are curated using Gblocks with a minimum block length of five positions, a maximum of 10 contiguous non-conserved positions, and considering only positions with a gap in less than 50% of the sequences in the final alignment. If the curation results are empty, the non-conserved alignment will be used for the tree. Finally, a maximum likelihood phylogenetic tree was inferred using FastTree43 v.2.1.10 from the curated amino acid alignment (Navarro-Muñoz et al., 2020).

#### BGC characterization of *Sphingomonas* genus

Name, source of isolation, country of isolation, genome size, and accessions were obtained from each representative complete genome of *Sphingomonas* species on the GenBank database. The genome in fasta format was analyzed by the antiSMASH v.5.1.0 bacterial version database to predict BGCs. AntiSMASH results were organized in a 49 × 19 matrix with the number of clusters and the corresponding type of the predicted metabolite. The genetic content for BGC on each genome was also calculated by the percentage of the base pair of the total BGCs divided by the genome size. Data were classified according to the source of the sample of each strain (water, soil, plant, animal, or unknown); and by extreme, mesophilic, or unknown environment, depending on the environmental conditions on the region of isolation.

#### Pangenome analysis

To unique genomic profile of the Antarctic strain and putative gene fission/fusion/duplication events were identified by pangenome analysis against representative genomes of *Sphingomonas*. Sequences annotated in Prokka were used as input on PIRATE v1.0.4 package (Bayliss et al., 2019). Default parameters were used with 60%, 70%, 80%, 90%, 90%, and 95% cutoff for amino acid identity. Briefly, PIRATE filters and reduces the dataset by iterative clustering using CD-HIT (Li and Godzik, 2006). The longest sequence from each CD-HIT cluster is used as a proxy for sequence similarity search with BLAST (Camacho et al., 2009). The normalized bit scores of the resulting all-*vs*-all comparisons are clustered using Markov Clustering Algorithm (MCL; Dongen, 2000) after eliminating matches below the percentage identity threshold. Unique clusters at higher thresholds are identified as “unique alleles.” In addition, a genomic island prediction was performed with IslandViewer v4 (Bertelli et al., 2017) and IslandCompare v1.0 (Bertelli et al., 2022) was used to identify the unique alleles associated with mobile elements among the closer strains.

## Results and discussion

### Genome-based taxonomic and phylogenetic analysis revealed that the Antarctic strain So64.6b might be a new species closely related to *Sphingomonas alpina*

**Figure 1:**
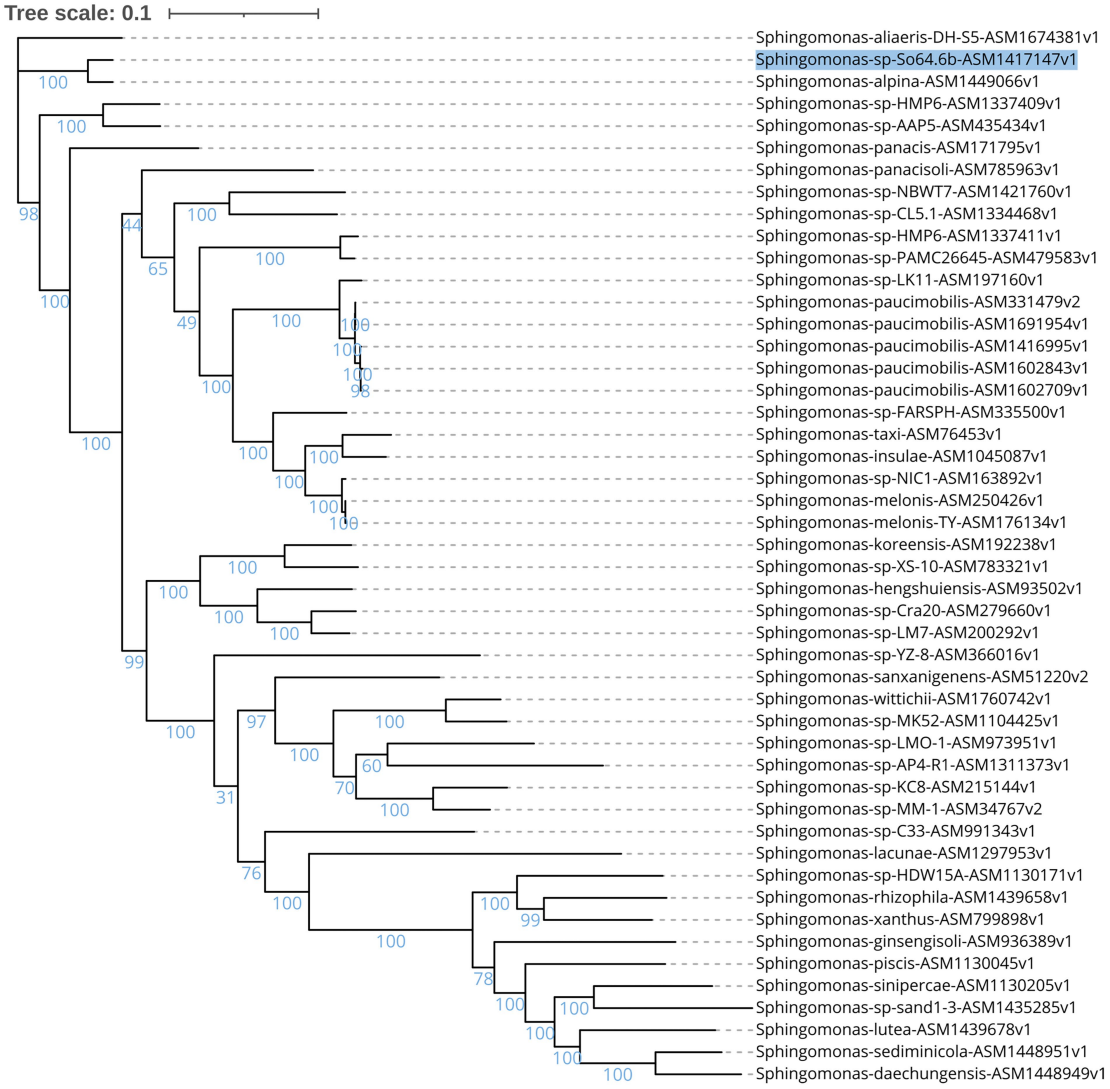
Maximun likelihood unrooted phylogenetic tree of proteome, based on sequences of concatenated amino acids from 126 ortholog genes present in 48 reference genomes from the *Sphingomonas* genus using RAxML. The accession number for the NCBI of each genome is present in the name. The branch lengths represent the number of substitutions per site (scale: 0.1 substitutions per site) and the percentage of the 1,000 replications bootstraps is also shown. Antarctic strain So64.6b is marked in blue.

The genome of the Antarctic strain So64.6b consists of 5,570,175 pb. Phylogeny based on core proteome showed that strain So64.6b is genetically close to *S. alpina,* followed by *Sphinogmonas* sp. strains AAP5 (isolated from alpine lake), HMP6 (isolated from ultra-oligotrophic lake in East Antarctica), and the species *S. aliaeris* DH-S5 (obtained from pork steak) and *S. panacis* (obtained from rusty ginseng root in South Korea based on Genbank repository records; (Singh et al., 2015; Sanangelantoni et al., 2018; Kim et al., 2020). The phylogeny shows a closer genetic relation of the Antarctic strain with *Sphingomonas alpina* DSM 22537 (S8-3T strain), a species isolated from the Alpes (Margesin et al., 2012; Figure 1). Both strains also share a 99.6% identity of 16S rRNA, assuming they belong to the same species. However, ANI values have no coincidence over the threshold that defines species (>95–96%) since they share 86% identity. Most similarities are between 76% and 78% with other species of *Sphingomonas*. (Supplementary Figure S1). The result confirms that *S. alpina* is the closest species, but *in silico* DDH calculates a 38% hybridization between *S. alpina* and our Antarctic isolate genome. Hence, our work suggests that S. *alpina* is a close relative of *Sphingomona*s sp. So64.6b, a potentially novel species of the genus *Sphingomonas.* Given the phylogenic results and to further explore the relation and differences that define each strain, we conducted an ortholog clusters analysis.

Both *Sphingomonas* strains shared 3,385 ortholog clusters, while 98 clusters were unique for the So46.6b strain and only 52 for the Alpes strain (Supplementary Figure S2). The Antarctic strain presented an enrichment of the transposition functions (value of *p* = 0.00076) and catabolism of aromatic compounds (value of *p* = 0.00019), while the Alpes strains have the greatest function in the oxidoreductase activity (value of *p* = 0.00061). Clusters on the So46.6b strain showed 10 transposases–grouped in two ortholog clusters– and four insertion elements related to genetic mobility. In addition, it contains multiple proteins for the catabolism of polluting agents such as benzoate, gallate, lignin, xylene, and tetrahydrofolate (Supplementary Table S1). That could be related to the presence of these phenolic pollutants in the Antarctic environments, where a higher rate of redox reactions on pollutants/heavy metals at a cold temperature might increase their harmful potential (Ju et al., 2017; Zangrando et al., 2019). Alpine strain does not have any of these genes and showed only proteins related to the catabolism of xylene and lignin, commonly found in the *Sphingomonas* genus (Menon et al., 2019). Therefore, we suggest genomic differences among the strains might be in part caused by a large accessory genome related with adaptation to the harsh Antarctic environment.

### BGCs of *Sphingomonas* sp. So64.6b might be related to the production of novel antibiotic molecules and showed evolutive divergences related to the isolation environment

The antiSMASH results are summarized in Table 1. *Sphingomonas sp.* So46.6b has a total of 6 BGCs related to polyketides synthases (PKS), terpene, two RiPP Recognition Elements-containing (RRE), lassopeptide, and a hybrid cluster lassopeptide-homoserine lactone. None of the BGCs have similarities to previously reported clusters from neither *Sphingomonas* nor other bacteria genres, excluding the terpene, which presented a low similarity (30%) to the known carotenoid BGC. This result indicates the potential of new untapped natural products from the Antarctic strain. Furthermore, BGCs prediction with the ARTS tool demonstrates that three of those clusters were also associated with the production of potentially novel antibiotic compounds due to their proximity with duplicated essential genes (×13), known resistant genes (×36), and recent horizontal gene transfer events (×189).

**Table 1:** Summary of Biosynthetic Gene Cluster (BGC) identified in the genome of Antarctic *Sphingomonas* sp. So64.6b and their genes related with auto-resistance to antibiotics when present on ARTS prediction.

Secondary metabolite (most similar known cluster)	From	To	Similarity to other clusters	Gene ID^a^	Description	Function
1: Lassopeptide-homoserinelactones	1,448,221	1,470,598	50% *Sphingomonas koreensis*	TIGR00224	pckA: phosphoenolpyruvate carboxykinase (ATP)	Energetic metabolism
2: RRE-containing	2,191,250	2,209,925	-	-	-	-
3: RRE-containing	2,287,599	2,306,484	-	-	-	-
4: Lassopeptide	3,477,189	3,499,720	23% *Sphingomonas leidyi*	TIGR00594	Polc: DNA polymerase III, alpha subunit	DNA metabolism
				TIGR02118	Conserved hypothetical protein	Hypothetical protein
5: T3PKS	3,889,391	3,930,452	51% *Sphingomonas melonis*	TIGR00357	Methionine-R-sulfoxide reductase	Cellular processes
				TIGR00621	ssb: single-stranded DNA binding protein	DNA metabolism
				TIGR00838	argH: argininosuccionate lyase	Aminoacids biosynthesis
				TIGR01026	flil_yscN: ATPase Flil/YscN family	Energetic metabolism
				TIGR01103	fliP: flagellar biosynthetic protein FliP	Cellular processes
				TIGR00797	matE: MATE efflux family protein	transport protein
6: Terpene (carotenoid, 30%)	4,072,179	4,096,869	-	-	-	-

a ID based on database of conserved proteins by a common ancestral sequence TIGRFAMs. – no matches on ARTS prediction.

Particularly we highlight the lassopeptide BGC-4 of *Sphingomonas sp*So64.6b as a potential novel antibiotic since it does not match with any other known BGC and has a low similarity with any other genetic cluster (Table 1, 23% shared genes with *S. leidyi*). Moreover, this cluster contains an essential gene related to resistance prediction due to duplication and Horizontal Gene Transfer (HGT) events, identified as a hypothetical protein (TIGR02118). A gene with this characteristic could be a resistant copy of the antibiotic target acquired by horizontal transfer along with the identified BGC (Alanjary et al., 2017). Most BGCs showed resistance genes related to typical functions of antibiotic activity, mainly in amino acid biosynthesis, protein production and transport, DNA synthesis, and central metabolism (Table 1). Although those BGCs might also produce unknown antibiotic compounds with usual targets, the BGC-4 cluster could be involved in more specific unknown activities; then, its lassopeptidic products could represent a new class of antibiotics with an unknown target molecule. The study of unknown targets for discovering new kinds of broad-spectrum antibiotics is considered one of the main strategies to fight against the antibiotic crisis (Matos De Opitz and Sass, 2020).

**Figure 2:**
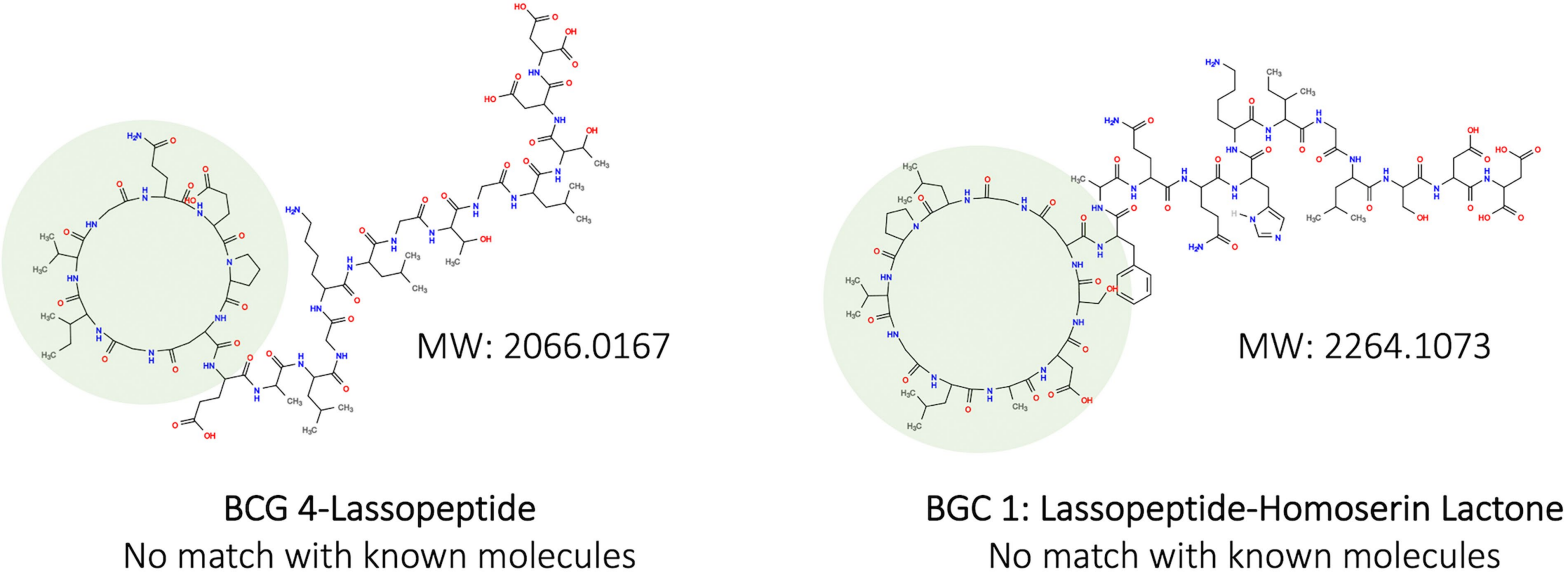
Predicted chemical structures produced by biosynthetic clusters in the genome of the Antarctic strain So64.6b based on PRISM detection. Macrocyclic ring formed by multiple N-terminal amino acid residues is highlighted in green. Remaining structure is the C-terminal tail of a lassopeptide. MW: molecular weight.

In addition, two molecular structures were predicted on PRIMS from the BGCs of So64.6b (Figure 2), one belonging to BGC-4. Both structures corresponded with lassopeptides types and showed high molecular weights (over 2,000 g/mol), which escapes from the detection spectrum of the antibiotic metabolites previously reported for this strain (Núñez-Montero et al., 2020). Such high molecular weight and predicted structure stand out the high molecular complexity of the compounds eventually produced by the BGC-4. This complexity in the lassopeptides structure appeals to their engineering regarding thermal stability, proteolytic protection, and chemical properties, promoting bioactive activities (Maksimov and Link, 2014).

Since we hypothesize that the Antarctic environment might be partially responsible for the unknown diversity of BGCs found on the strain So64.6b, a phylogenetic analysis using CORASON was performed to identify genetic divergences of a common BGC between the Antarctic bacteria and other closely related *Sphingomonas* strains. This analysis is an alternative for prioritizing potentially novel BGCs based on comparative phylogenomics. Instead of comparing between full and partial merged BGCs as done with other tools, CORASON avoids artifact errors by a glocal-type comparison, which first finds the longest common subcluster between the PFAM of a pair of BGCs (including our Antarctic query strain) and continues with an extension of this alignment applying match/mismatch penalties (Navarro-Muñoz et al., 2020). The analysis was performed using the BGC-6 of strain So64.6b since this was the only cluster shared with other *Sphingomonas* species, including Antarctic strains, and matching a known BGC on MiBIG database. The synteny of genes was based on the carotenoid cluster, specifically by comparing the gene *ctrl*, coding for the phytoene desaturase enzyme. This enzyme converts phytoene or squalene to lycopene by desaturation in four places.

**Figure 3:**
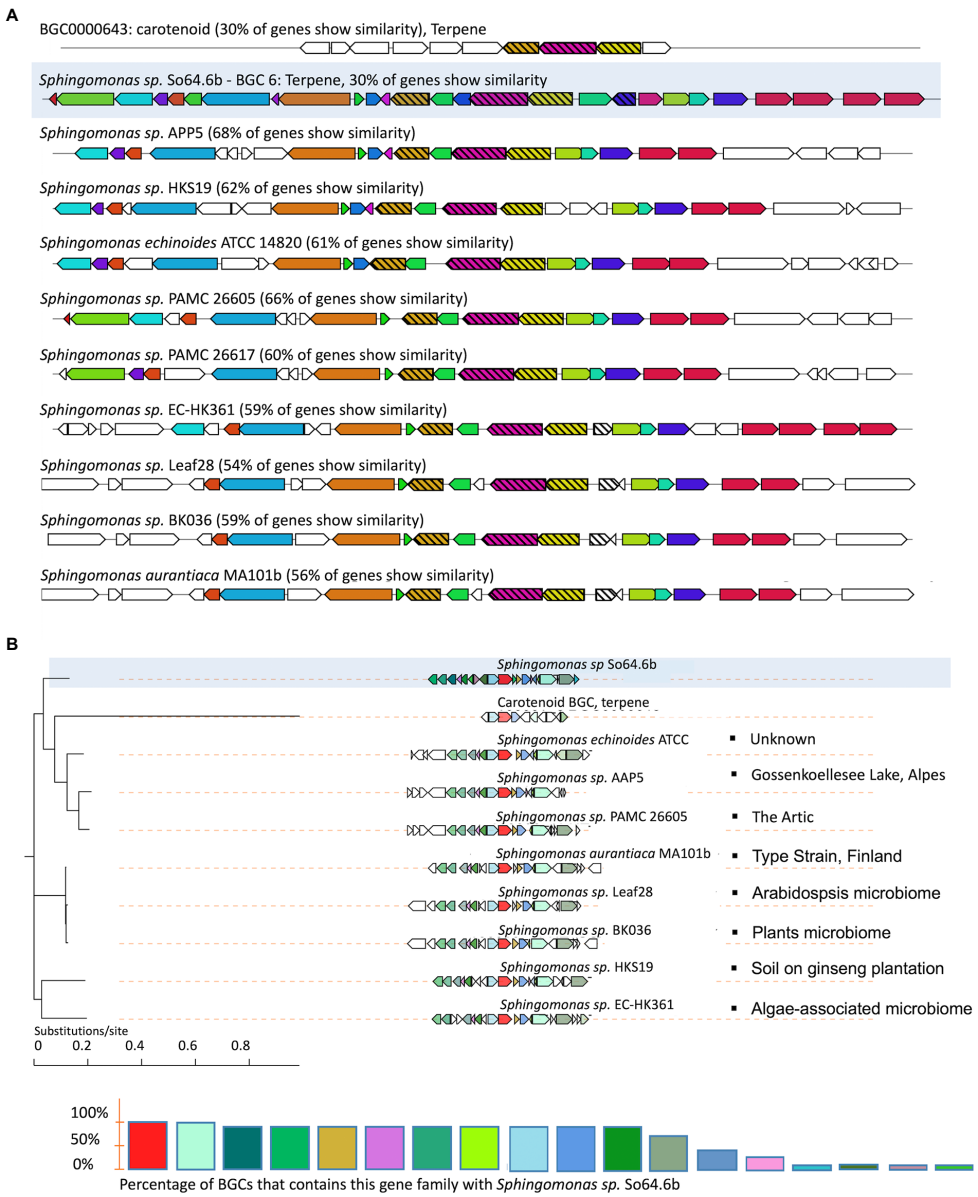
Variation in the genomic proximity of the terpene-like BGC-6 of the Antarctic strain So64.6b with similarity to the MiBIG reference BGC0000643 from *Brevundimonas vesicularis* (Tao et al., 2006). **(A)** Similarity plots of terpene BGCs from species of the genus *Sphingomonas*, including the Antarctic *Sphingomonas sp*. So64.6 (highlighted) and its closest species, mapped to the known carotenoid cluster BGC0000643 (on top). The colors match the shared genes, and those related with biosynthesis are shown in strip-pattern. **(B)** Terpene cluster phylogeny inferred by CORASON showing the frequency of occurrence of each gene family in comparison with the cluster from Antarctic strain So64.6b. Differences in sequence similarity are also indicated by a color gradient in the depicted gene blocks. The bullets at the end of each branch indicate the bacterial origin according to the metadata reported for the sample (Biosample data from NCBI database).

The result shows a unique and shared syntenic organization of the BGC across nine strains of the genus *Sphingomonas,* where 54–68% of the genes were found in all the strains (Figure 3A). Furthermore, the phylogenetic result showed a clear grouping of the cluster in three clades, with the Antarctic strain cluster being the most distant one from the rest of the sequences (Figure 3B). In this context, it is interesting to notice that the Antarctic So64.6b strain shares a clade with strains from similar extreme environments, including the PAMC strain obtained from the Arctic (Shin et al., 2012) –and the AAP strain, isolated from an Alpine lake (Kopejtka et al., 2020). Such environments shared extreme conditions such as high altitude, high UV radiations and exposure to freeze/thaw cycles. Besides, strains related to different environments, such as those isolated from plant microbiome or soil, are grouped apart from those of extreme environments (Figure 3B). The production of pigments has been reported in other Antarctic organisms to cope with high UV radiation (Rodrigues et al., 2021; Singh et al., 2022). Then, this carotenoid variations might play a role on the resistance to UV radiation. The result supports our hypothesis showing a genetic divergence on an Antarctic strain’s BGC; while such genetic differences might drive the variations observed in the biosynthetic modules, highlighting its potential for finding new molecules. In addition, that genetic divergence could be associated with the adaptation of this bacteria to an extreme environment. Specialized metabolites have evolved to mediate important ecological functions, explaining their diversity of chemical products (van Bergeijk et al., 2020). Our hypothesis established that some of those metabolites might be driven by the Antarctic environmental harsh conditions. Other authors have reports findings that are consistent with this theory. Among then, chromomycin, an antibiotic produced by *Streptomyces flaviscleroticus* showed antioxidant effect that confers resistance oxidative stress (Prajapati et al., 2019); the lipid-based esters, and Cys-GSH isomers were related to antioxidant response, significantly shifted by Cadmium concentrations in *Scenedesmus obliquus* (Mangal et al., 2020); secondary metabolites production and additional genes for specific peptides were found to potentially enhance the response to environmental changes on members of the order *Candidatus Thermoprofundales*, enabling them inhabit marine hydrothermal vents, deep subsurface oil reservoirs and hot springs (Liu et al., 2022); and the products of shikimate pathway including mycosporines and mycosporines-like-amino-acids which are well-known radiation resistant substances (Sarkar et al., 2018; Gacem et al., 2021).

**Figure 4:**
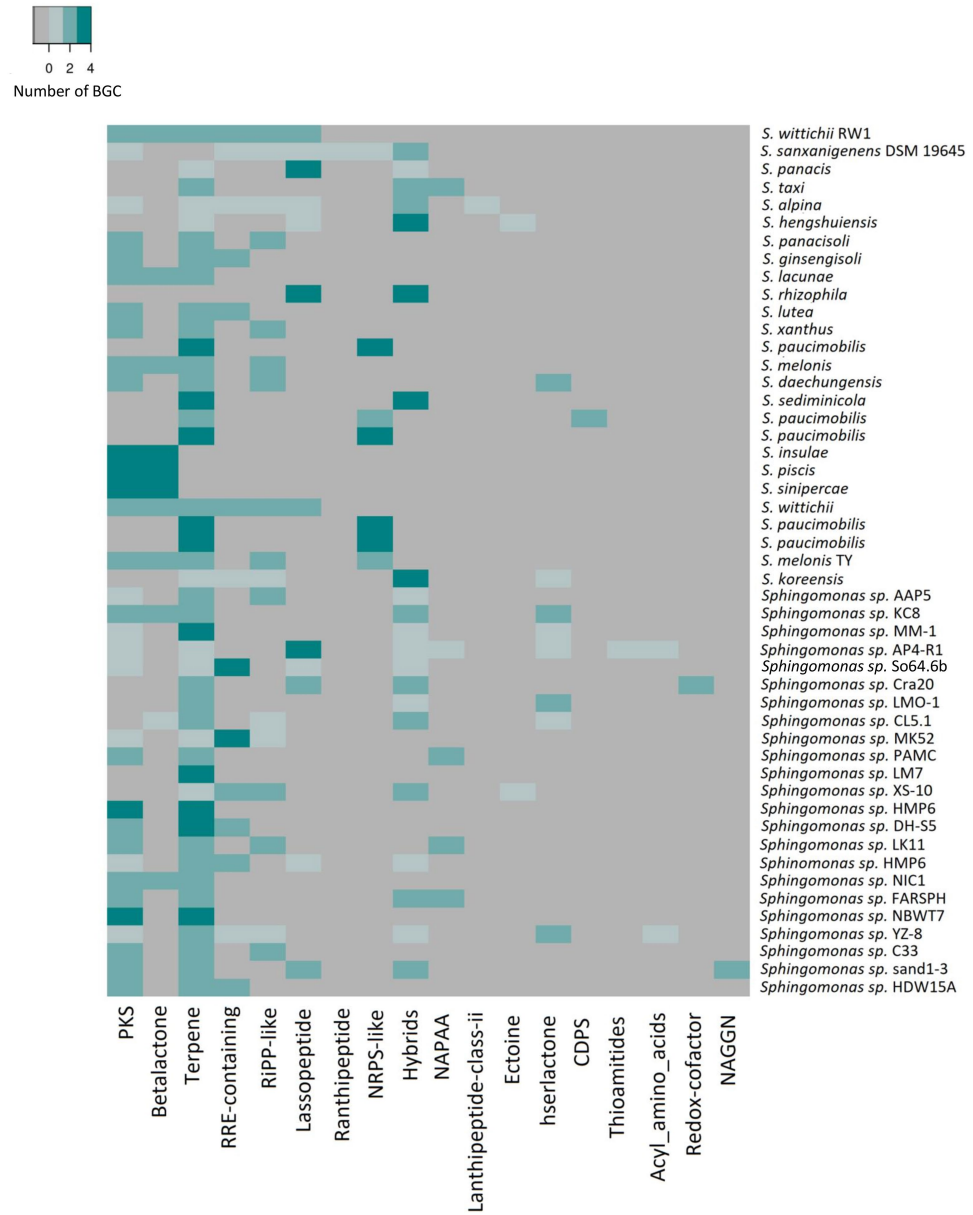
Heatmap of number and types of BGCs predicted by AntiSMASH analysis for 49 representative genomes of *Sphingomonas* genus, including the Antarctic strain So64.6b. The color scale represents the number of counts per type of BGC found on the genome.

To further support this idea we checked whether the environmental conditions influences the overall composition and diversity of BGCs on *Sphingomonas* strains by comparing the predicted BGCs within this genus and between the representative genomes of *Sphingomonas* species isolated from different environments available on the Genbank database. An average of four clusters were found on 49 genomes analyzed, ranging from one in *Sphingomonas* sp. LM7, to eight in *Sphingomonas sp.* AP4-R1 (Figure 4). Previous studies regarding genome-mining of proteobacteria species also showed that the most common types of BGC were nonribosomal peptide synthetases (NRPS), PKS, and hybrids; however, lanthipeptides and terpenes are also present (Brotherton et al., 2018). NRPS and PKS are peptide biosynthesis machinery of natural products with multiple bioactivities (Giordano et al., 2015). Likewise, lanthipeptides and terpenes have been related to antimicrobial activity compounds (Giordano et al., 2015; Oladipo et al., 2022).

**Figure 5:**
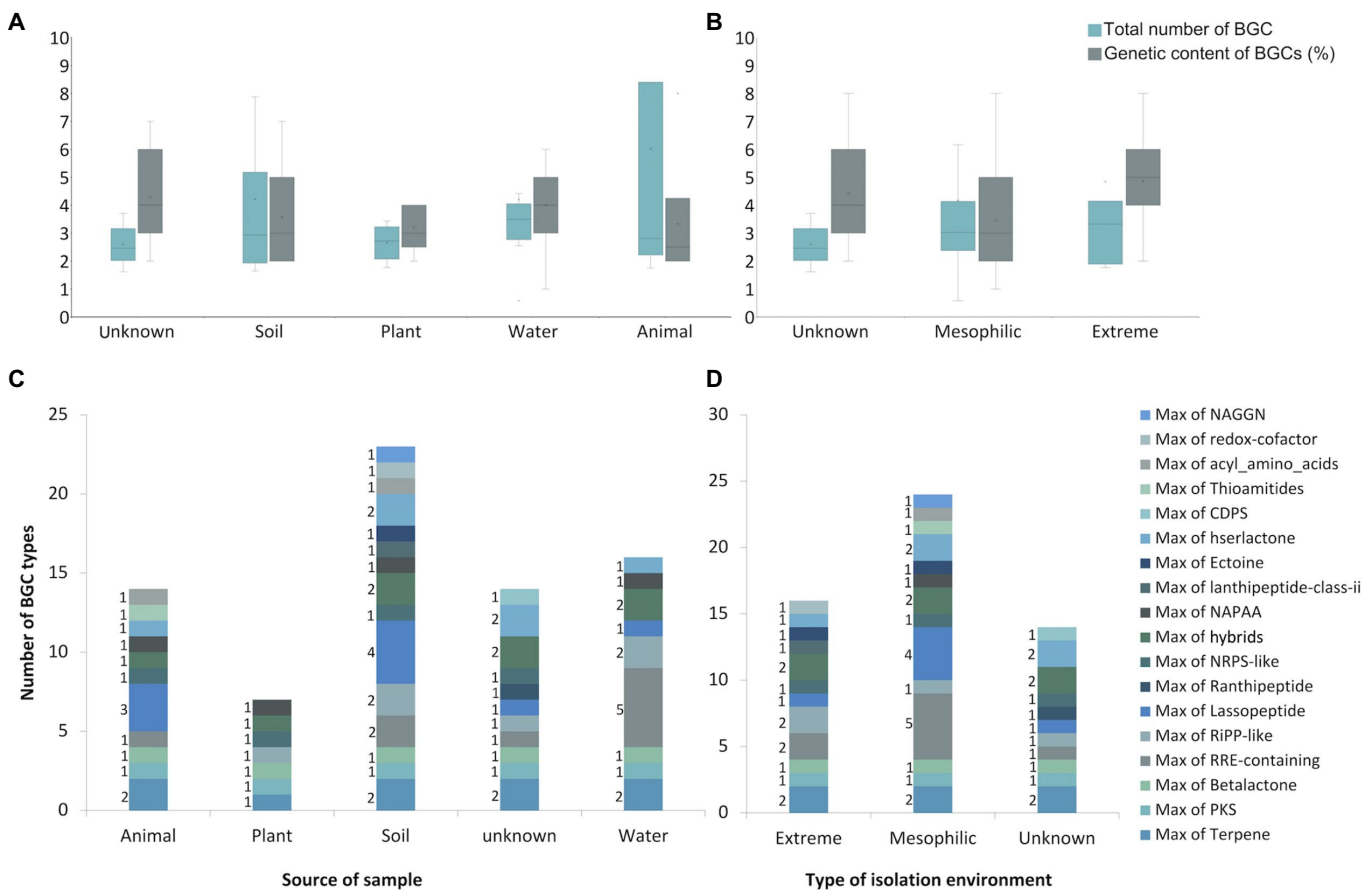
BGCs characterization of the *Sphingomonas* genus. **(A)** The Maximum number of BGC metabolites by the source of samples. **(B)** The Maximum number of BGC metabolites by environmental conditions. **(C)** Genetic content for BGCs and number of clusters by the source of the sample. **(D)** Genetic content for BGCs and number of clusters by environmental conditions. In C and D box plot are shown, where central lines of the box represent the median derived using the lower and upper quartile values. The maximum and minimum values are displayed with vertical lines connecting the points to the center box. NAGGN: N-acetylglutaminylglutamine amide dipeptide. CDPS: cyclodipeptides. NAPAA: Non-Alpha Poly-Amino group Acids. NRPS: Non-Ribosomal Peptide Synthetase. RiPP: Ribosomally synthesized and Post-translationally modified Peptides. RRE: RiPP precursor peptide Recognition Element. PKS: Polyketide Synthases.

The total amount of clusters in the strains was variable; then, to understand whether it can be affected by the origin of each sample, they were grouped by source of isolation from animals (×6), plants (×5), soil (×19), water (×12) or from an unknown source (×7) when it was not reported on Biosample Genbank information. The samples were also classified into extreme or mesophilic environments regarding the expected conditions of the region of the isolation source. Strains from water showed the greatest average of the total amount of predicted clusters (×4), while animal-isolated bacteria were the lowest. Nonetheless, soil isolated bacteria have the maximum number of clusters (×8), while plant isolated bacteria showed the lowest maximum (×4). In addition, animal-isolated bacteria had the greatest genetic content devoted to BGC in their genome (6.02%) compared to strains isolated from plants (2.66%), soil (2.60%), and water (4.20%; Figure 5C). This result suggests that the total amount of BGCs in the *Sphingomonas* might be related to the conditions they face in their specific samples. Also, strains from animals seem to have fewer BGCs, but with more genetic content on each cluster, which could be associated with a stronger regulation due to the interspecies communication or additional biosynthetic genes for modifications on the scaffold molecule to produce a larger number of derived products. Besides, soil and water samples were related to a larger amount of BGCs, mainly NRPS, PKS, and ribosomally synthesized and post-translationally modified peptides (RiPPs). These results are similar to other studies on *Streptomyces* mining for specialized metabolism and marine *Pseudoalteromonas* pangenome analysis (Cordero and Polz, 2014; Maansson et al., 2016; Nicault et al., 2020)

In the environment-type context, seven bacteria were classified as isolated from extreme environments, 35 strains were isolated from mesophilic environments, and seven could not be classified. Bacteria from extreme environments had the greatest average of BGC (five), while mesophilic environments had the lowest (three). However, the same maximum number of clusters (eight) were found on strains from all environments. Interestingly, bacteria isolated from extreme environments also presented the greatest average of genetic content devoted to BGC (4.84% vs. 4.17% in mesophilic environment, Figure 5D). The results also indicate a major capacity for production of secondary metabolites in *Sphingomonas* isolated extreme environments, compared to the average percentage of 3.7% (± 3.1%) reported for proteobacteria phyla (Cimermancic et al., 2014).

To explore whether those differences were also found for particular types of BGCs –because of their different functionalities– the amount of each type of BGCs across the genomes was compared and related to the isolation origin. More than 16 types of BGCs were found across the *Sphingomonas* genomes. Interestingly, a maximum number of five clusters associated with the type of RRE-containing metabolites were found on species isolated from water. Conversely, other BGC types were less commonly found in the sum of similar samples, and, particularly, the RRE-containing type was present in less than two isolated species in other types of samples. In contrast, the plant isolated species did not have this type of BGC (Figure 5A). Besides, a higher number of lassopeptides-related clusters were found in soil and animal isolated species (four and three, respectively); one was identified in water and an unknown source, while none was present for plant isolated species (Figure 5A). In addition, the greatest amount of lassopeptides and RRE-containing metabolites were found in mesophilic environments (five and four, respectively). On the other hand, strains from extreme environments exclusively present clusters related to lanthipeptide class II and redox cofactor (Figure 5B). These can be associated with the adaption to the oxidative stress and low-temperature conditions of the Antarctic environment (Simpson and Codd, 2011; Grimalt-Alemany et al., 2021). The overall results showed that the number and some types of BGCs found on *Sphingomonas* genus seems to be associated with the environment they inhabit. Then BGC content in thus genus might be drive as evolutionary response to the requirements of the specific environment. Particularly, the identified BGCs in our soil-extreme environment bacteria might be related to an ecological advantage instead of taxonomic characteristics. This is expected since in those environments, bacteria deal with multiple physico-chemical complex changes, requiring a plethora of physiological and metabolic response (Cordero and Polz, 2014). Here we highlight the relevance of elucidating new natural products from strain So64.6b –isolated from polyextreme soil–, as others of biosynthetic interest in the *Sphinogmonas* species.

### Pangenome analysis showed mobile genetic elements and resistance genes associated with the adaptation of *Sphingomonas* sp. So64.6b to the Antarctic environment

Since the Antarctic strain So64.6b had characteristics related to the metabolite production potential, we continue to study if this strain has other genetic elements particularly related to its adaptability to the Antarctic environment explaining its divergent genome content. From the pangenome analysis of the *Sphingomonas* genus, 34,242 genetic families were obtained, 1,546 were unique for the Antarctic strain, including metabolic pathways for the degradation of aromatic compounds previously mentioned. The result evidenced a significant genetic diversity of the genus with a vast number of unique genes belonging to one species. This is consistent with similar studies on comparative genomics of *Sphingomonas*, where it has been suggested that the presence of this group in a wide diversity of environments has led to a large genetic difference and a small core genome for the genus (Kim et al., 2020).

We identify multiple ribonucleases, duplicated genes for the synthesis of asparagine, kinases, and aminotransferases of serine exclusively present on the genome of the Antarctic So46.6b strain. Considering that a great genetic variability might be associated with the adaptation mechanisms of *Sphingomonas* species to specific conditions, this can be part of adaptation to extreme temperatures. For example, it has been described that highly catalytic enzymes at low temperatures have a greater amount of asparagine and serine than their mesophilic homologs, with more arginine and lysine (Qin et al., 2014).

In addition, the Antarctic strain shows multiple unique genes related to antibiotic and heavy metal resistance, such as *CzcC* protein for cobalt, zinc, and cadmium resistance; *CorA* for cobalt and magnesium transportations; copper resistance and transportation proteins; receptors and iron transporters (*FepC* and *FieF*); regulators for the resistance and reduction of mercury; metalo-beta-lactamases; multidrug excretion pumps (*EmrA, EmrB, AcrB, MedtA-C-G, MexA, NorM, Stp, OqxB13-17); SugE* for quaternary ammonium resistance, and penicillin-binding proteins. These genes might also have an important role in the Antarctic environment. Studies have reported the presence of pollutants in the Antarctic continent, including heavy metals (cadmium, zinc, vanadium, arsenic, and gold), antibiotics, and pesticides transported by natural processes through the air and water (Corsolini, 2009; Lo Giudice et al., 2013). Also, in this place, active volcanoes contribute a significant amount of heavy metals to the soil (Singh et al., 2007). Regarding antibiotic resistance, it has been described that the introduction of foreign microorganisms due to tourism and scientific activities increased the acquisition of these genes by HGT (Cowan et al., 2011).

**Figure 6:**
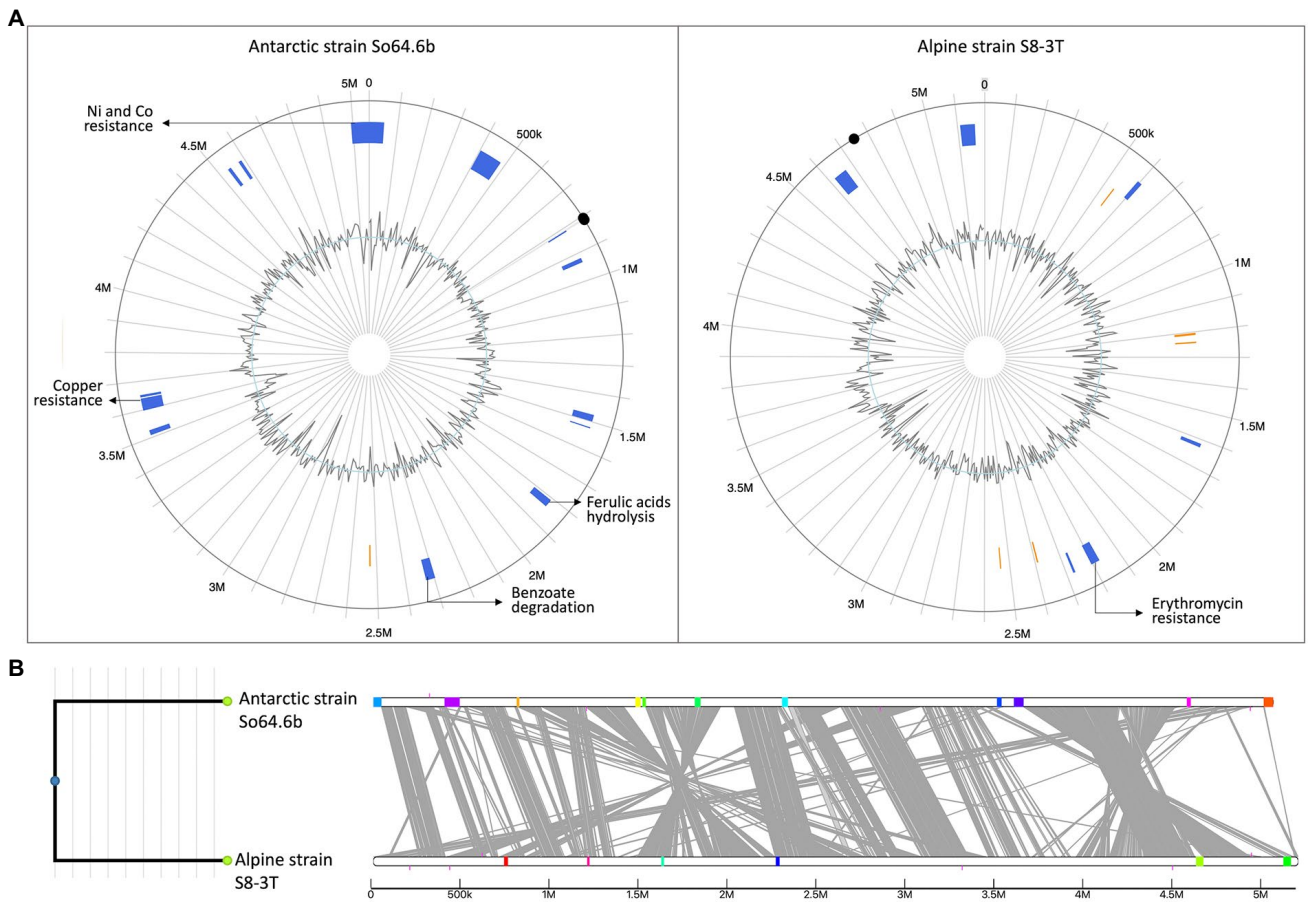
Genomic island (GIs) predictions and locations in two *Sphingomonas* genomes. **(A)** GI predictions using Island Viewer 4 and the two circular genomes of Antarctic Sphingomonas strain So64.6b (left) and Alpine strains *S. alpina* S8-3T (right). The circle represents a single chromosome, within the circle, GIs predictions are marked as colored blocks according to the prediction method; IslandPath-DIMOB (blue) and SIGI-HMM (orange). Evidence of genes related with adaptability, metabolism or resistances are described on pointing arrows. Inner line shows the change in GC skew. Black dots mark the OriC, predicted by proximity with *recA*, *rnpA* and *dnaA* and verified with Ori-Finder tool (Dong et al., 2022). **(B)** Comparative genomics visualization the *Sphingomonas* genomes. GIs are represented as coloured blocks placed on a linear representation of the genome (linear white bars indicate genomes, with alignments between genomes shown in grey). Same color represents matching GIs.

Our results highlight the genetic dynamism of the *Sphingomonas* genus. Specifically for the Antarctic strain, we reported enrichment of ortholog genes related to transposons and resistance genes, usually transferable by plasmids, and pointed out multiple resistance genes. To confirm whether those unique genes of the Antarctic strain could have been acquired by mobile elements, driving the genetic differences among the Antarctic strain and the Alpine strain (closer relative), we studied the genomic islands in its genome. More genomic islands were identified on the genome of the Antarctic strain (Figure 6A) and most of them were unique for this genome (Figure 6B). Within those regions, we identified the unique genes of this strain related to degradation of benzoate, hydrolysis of ferulic acids, resistance to copper, nickel, and cobalt, as well as multiple transposases and insertion elements. Meanwhile, genomic island on Alpine strain were mostly composed of phage genes, transposases or hypothetical proteins. Some were already present in the Antarctic strain and only one gene of antibiotic resistance were found (erythromycin esterase) as evidence of adaptability genetic material. Hence these results suggest that the differential genetic elements of the Antarctic strain So64.6b could be recently acquired by HGT environments to cope with the Antarctic poly-extreme conditions. Previous studies regarding genetic diversity showed a correlation between taxonomic distance and the production of secondary metabolites featuring unique bioactive natural products (Hoffmann et al., 2018). Also, similar results were observed in *Pseudoalteromonas* sp., a model of adaptation to extreme environments. A notable genomic diversity on this group has been attributed to recent acquisition a large set of mobile elements, mainly by HGT (Parrilli et al., 2021). The results support our hypothesis that some of the BGCs detected in this strain might be linked to the adaptation to harsh conditions of the Antarctic; for example, those without similarity to any other clusters within its genus or phylogenetically closer to strains from similar environments. Therefore, the *Sphingomonas sp.* So64.6b strain should be considered a new source for mining and bioprospecting antimicrobials compounds.

Strains belonging to the family Sphingomonadaceae have been explored due to their biotechnological applications in bioremediation and degradation of refractory pollutants and in the production of valuable biopolymers called sphinganes (Huang et al., 2009), which have an important role as growth promoters in plants. Particularly the genus *Sphingomonas* has been characterized as a degrader of multiple environmental pollutants, so, in addition to its role as a soil conditioner, it has a relevant ecological role in the recycling of complex molecules (Bhatt et al., 2020; Zhang et al., 2020). Also, due to its capacity to store heavy metals, it has improved plant resistance in environments with high zinc, cadmium, and copper contents, in addition to collaborating in stress conditions such as drought (Asaf et al., 2020). This background agrees with the genomic results observed in the Antarctic strain, showing a high capacity for resistance to metals and degradation of pollutants, which appear to be unique to the strain and absent even its closest relative. This highlights the influence that the selective pressure of the Antarctic environment may have exerted on the evolution, shaping a likely new species; and the genetic conformation of the Antarctic strain under study related with an essential role it probably plays in the Antarctic soil by protecting other organisms from harmful compounds. Moreover, the high genetic variation observed in this work, which has been previously reported for the genus *Sphingomonas*, together with the unique BGCs, make this Antarctic strain an important target in the searching for new metabolic pathways and the discovery of natural products.

## Data availability statement

The datasets presented in this study can be found in online repositories. The names of the repository/repositories and accession number (s) can be found in the article/Supplementary material.

## Author contributions

KN-M: conceptualization, methodology, validation, formal analysis, data curation, writing—original draft preparation, writing—review and editing, and visualization. DR-V: formal analysis, data curation, writing—original draft preparation, writing—review and editing, and visualization. LB: conceptualization, supervision, project administration, and funding acquisition. All authors contributed to the article and approved the submitted version.

## Funding

This research was funded by grants from the Agencia Nacional de Investigacion y Desarrollo de Chile (ANID) - FONDECYT-1210563, the Instituto Antártico Chileno (INACH), INACH DG_01–19; Network for Extreme Environments Research (NEXER), NXR17-0003; INSTITUTO TECNOLÓGICO DE COSTA RICA project 5402–1510-1035, and CONICYT–PFCHA/Doctorado Nacional/2017–21170263 for KN-M.

## Conflict of interest

The authors declare that the research was conducted in the absence of any commercial or financial relationships that could be construed as a potential conflict of interest.

## Publisher’s note

All claims expressed in this article are solely those of the authors and do not necessarily represent those of their affiliated organizations, or those of the publisher, the editors and the reviewers. Any product that may be evaluated in this article, or claim that may be made by its manufacturer, is not guaranteed or endorsed by the publisher.

## Footnotes

^1^ https://microbializer.tau.ac.il/

^2^ https://bigscape-corason.secondarymetabolites.org
