# Supplementary material for: Antarctic Sphingomonas sp. So64.6b showed evolutive divergence within its genus, including new biosynthetic gene clusters
Source: Front Microbiol. 2022 Nov 18;13:1007225. doi: 10.3389/fmicb.2022.1007225 (PMC9716278; doi:10.3389/fmicb.2022.1007225)
Supplement: Supplementary file 1 [file Data_Sheet_1.docx]

Supplementary Material

**Supplementary table 1**. Principal enriched unique cluster found in the Antarctic strain *Sphingomonas sp.* So64.6b by comparative analysis of genetic orthology, including clusters related to transposition and degradation of aromatic compounds.

| Cluster | Proteins | | | Swiss-Prot ID | | Annotation GO | |
| --- | --- | --- | --- | --- | --- | --- | --- |
| cluster2 | 6 | | | P0CF79: Transposase InsF | | GO:0032196; P: transposition; IDA:EcoCyc | |
| cluster10 | 4 | | | Q46087: Transposase nmoT | | GO:0032196; P: transposition; IEA:UniProtKB-KW | |
| cluster19 | 4 | | | Q44256: 3-chlorobenzoate-3,4-dioxygenase oxygenase subunit cbaA | | GO:0019439; P: aromatic compound catabolic process; IEA:UniProtKB-KW | |
| cluster21 | 4 | | | P16939: Insertion element IS600 | | GO:0006313; P: transposition, DNA-mediated; IEA:InterPro | |
| cluster29 | 3 | | | P80402: 2,3-dihydroxybenzoate decarboxylase | | GO:0043640; P: benzoate catabolic process via hydroxylation; IEA:UniProtKB-UniPathway | |
| cluster33 | 3 | | | P30347: Protein LigF | | GO:0046274; P: lignin catabolic process; IEA:UniProtKB-KW | |
| cluster39 | 3 | | | Q6N8N2: Cytochrome p450 CYP199A2, oxidation of methoxybenzoate | | GO:0006805; P: xenobiotic metabolic process; IDA:UniProtKB | |
| cluster76 | 2 | | | Q0CBM8: Probable endo-1,4-beta-xylanase C | | GO:0045493; P: xylan catabolic process; ISS:UniProtKB | |
| cluster85 | 2 | | | P22635: Protocatechuate 4,5-dioxygenase alpha chain ligA | | GO:0019439; P: aromatic compound catabolic process; IEA:UniProtKB-KW | |
| cluster102 | 2 | | | A5V4U1: Bifunctional protein FolD 1 | | GO:0035999; P: tetrahydrofolate interconversion; IEA:UniProtKB-UniPathway | |
| cluster104 | 2 | | | Q9HUR2: Benzoylformate decarboxylase mdlC | | GO:0019596; P: mandelate catabolic process; IEA:UniProtKB-UniPathway | |
| cluster118 | 2 | | | P22636: Protocatechuate 4,5-dioxygenase beta chain ligB | | GO:0019439; P: aromatic compound catabolic process; IEA:UniProtKB-KW | |
| cluster128 | | 2 | Q88JX9: 4-carboxy-4-hydroxy-2-oxoadipic acid aldolase galC | | GO:0019396; P: gallate catabolic process; IDA:UniProtKB | |  |

**Supplementary Figure 1.** ANI value calculated by the BLASTn method of the *Sphingomnas* strains. A) Neighbor joining dendrogram based on ANI values of genomic sequences showing the position of the Antarctic strain and the most related taxa. Bar, 10% difference in ANI value. B) Heatmap matrix of ANI aggrupation between the 48 reference genomes of the *Sphingomonas* genre, including the So64.6b Antarctic strain the most related taxa (highlighted). The accession number for each GenBank genome are indicated in the name.


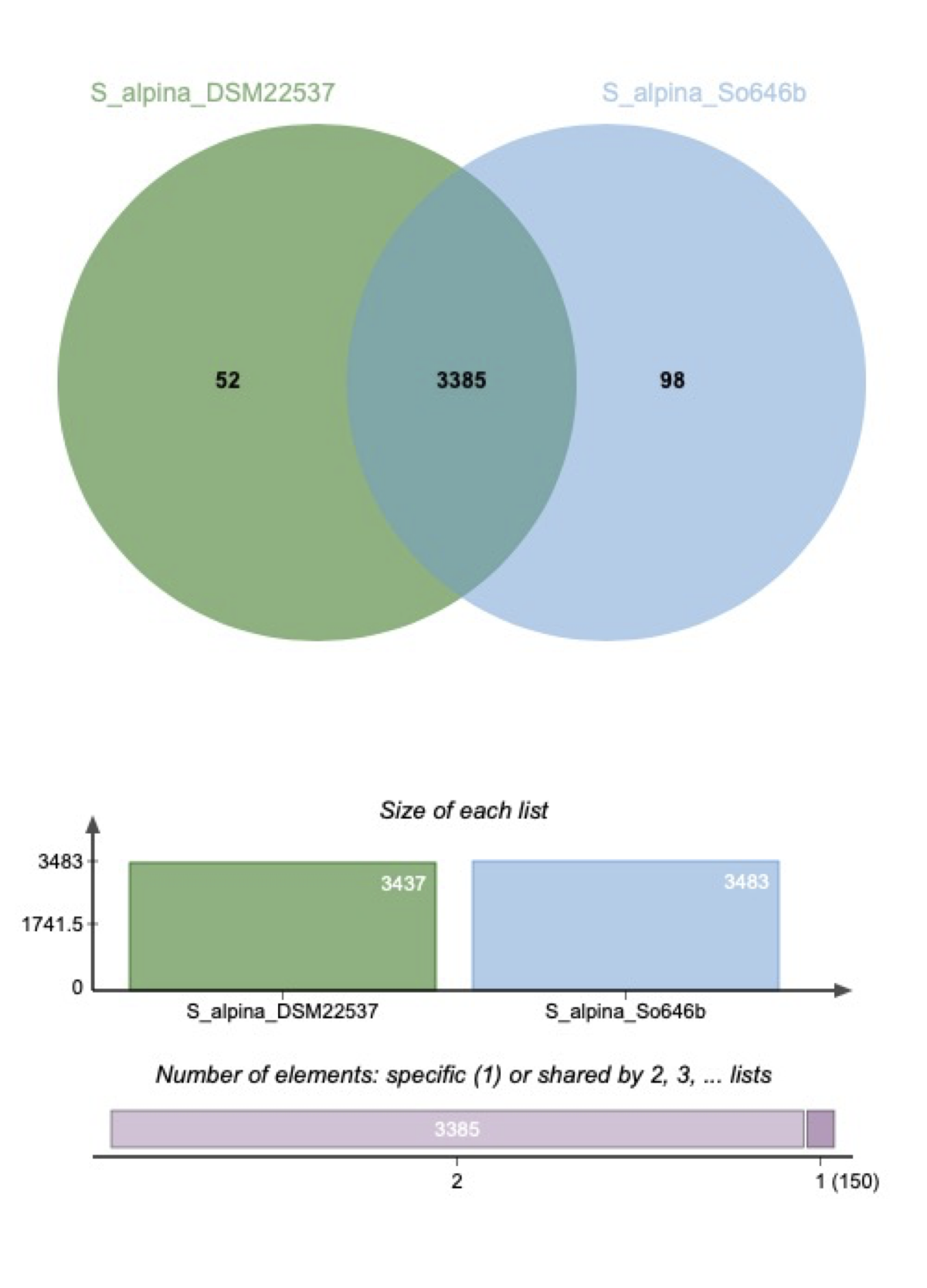


**Supplementary Figure 2.** Venn’s diagram comparing the distribution of ortholog cluster between the two *Sphingomonas alpina* and the Antarctic strain *Sphingomonas* sp. So64.6b.
